# Inequalities in the prevalence of double burden of malnutrition among mother–child dyads in India

**DOI:** 10.1038/s41598-023-43993-z

**Published:** 2023-10-07

**Authors:** Saurabh Singh, Neha Shri, Akancha Singh

**Affiliations:** International Institute for Population Sciences, Mumbai, 400088 Maharashtra India

**Keywords:** Public health, Epidemiology

## Abstract

In the midst of rapid urbanization and economic shifts, the global landscape witnesses a surge in overweight and obese individuals, even as child malnutrition persists as a formidable public health challenge in low- and middle-income countries (LMICs). This study seeks to unravel the prevalence of the double burden of malnutrition (DBM) within the context of India and delve into the associated disparities rooted in wealth. This study leverages data from the fifth wave of the National Family and Health Survey (NFHS-5), a nationally representative survey conducted in the year 2019–21 in India. This study focuses on mother–child dyads with children under the age of 3 years. Descriptive, bivariate and logistic regression analysis is used to decipher the intricate web of DBM’s prevalence and risk factors, as underscored by socio-demographic attributes. Wagstaff decomposition analysis is applied to quantify the contribution of each inequality in the social determinants on the observed income-related inequality in the DBM. Result from bivariate and logistic regression indicated a heightened risk of DBM within households marked by C-section births, affluence, ongoing breastfeeding practices, advanced maternal age, and larger household sizes. Additionally, households harbouring women with abdominal obesity emerge as hotspots for elevated DBM risk. Notably, the interplay of abdominal obesity and geographical disparities looms large as drivers of substantial inequality in DBM prevalence, whereas other factors exert a comparably milder influence. As India grapples with the burgeoning burden of DBM, a conspicuous imbalance in its prevalence pervades, albeit inadequately addressed. This juncture warrants the formulation of dual-purpose strategies, and a slew of innovative actions to deftly navigate the complex challenges poised by the dual burden of malnutrition. Amidst these exigencies, the imperative to forge a holistic approach that encompasses both sides of the malnutrition spectrum remains a beacon guiding the quest for equitable health and nutrition outcomes.

## Introduction

One of the most challenging public health issues in low and middle-income countries is the problem of maternal and child malnutrition^1^. The double burden of malnutrition (DBM) is said to exist when there is a coexistence of maternal overweight/obesity and child undernutrition in the same household^2^. The coexistence of overnutrition and undernutrition exacerbates the risk of several health issues^3^. Undernutrition places children at the risk of childhood mortality and hindered cognitive growth^4^, while over-nutrition in women is associated with an increased risk of various non-communicable diseases such as diabetes, hypertension, increased lipid profiles, and abdominal obesity^5^. Being overweight/obese, especially during pregnancy, is proven to have a positive association with many unfavorable maternal and fetal outcomes during pregnancy, delivery, and postpartum^6^.

The global prevalence of stunting, wasting, and underweight among children has reduced substantially from an estimated 40 percent in 1990 to 26 percent in 2011, with an average annual rate of reduction of 2.1 percent^3^. This has happened concomitantly with an estimated increase of 10 percent in the proportion of overweight mothers between 1990 and 2008, while an increase of 5 percent was also registered in maternal obesity during the same period^3^. Additionally, it is projected that the prevalence of overweight and obesity in South and Southeast Asia will increase by two-thirds by 2030^6^.

Previous literature suggests that overweight and obesity are important risk factors for overall mortality^7^, chronic diseases such as cardiovascular diseases^8^, diabetes^9^ multimorbidity^8,10^, and disabilities^11^. Along similar lines, being underweight strongly predicts premature mortality, poor self-rated health, well-being, and disabilities. This association is robust in developing countries^12,13^.

Research suggests that a combination of maternal overweight/obesity and child undernutrition is the result of an interaction of a gamut of factors such as the socio-economic status of the household, dietary habits, and intensity of physical activities^14^. Additionally, most low and middle-income countries are experiencing economic and nutrition transitions^15^. The factors associated with the DBM are maternal age, maternal height, maternal education, and household wealth^16–18^.

**Figure 1:**
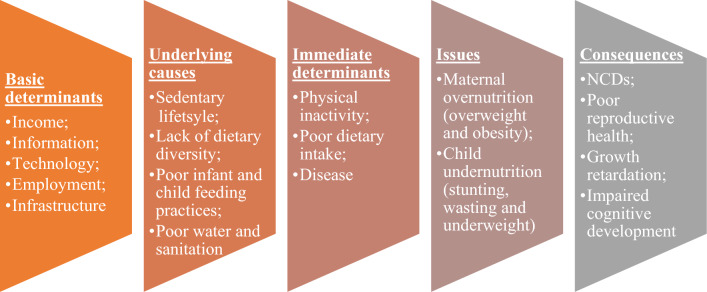
Conceptual framework for the determinants of DBM among mother–child dyads. Source: Adapted from WHO (2016).

Studies on DBM in India have primarily focussed on individual population groups, such as adults^19^, adolescents^20^, and women^21^. However, limited studies have tried addressing DBM in India's mother–child dyads^22,23^. Studies that have addressed the above have is limited to individual states such as Delhi^23^ and Kerala^22^. Evidences show that the poor are disproportionately affected by maternal and child undernutrition^24,25^, especially urban poor groups where undernutrition and other health issues are worse than among their richer counterparts^26,27^. A study conducted by Nguyen and colleagues reported that wealth inequalities existed largely for stunting within residence among children in India however, there has been a rise in the overweight/obesity among women specially among those residing in rural area and Urban slum. The same study also highlighted that within-residence, wealth inequalities were large for both underweight and overweight/obesity for adults, with the former being more concentrated among poorer households and the latter among wealthier households^28^. Moreover, decreasing trends in underweight and increasing trends in overweight prevalence in the period 2000–2017 has been observed in Southeast Asian countries including Bangladesh and India^29^. Previous studies have stated that it is crucial to acknowledge the coexistence of undernutrition, obesity, and non-communicable diseases, especially in low and middle-income countries, because these often tend to occur in the same social stratum^30^. While previous research has explored DBM in various demographic groups, this study specifically targets mother–child pairs, providing a holistic perspective on how maternal and child nutritional statuses intertwine using nationally representative data. By investigating both mother and child within the same household and dissecting how socioeconomic factors influence the likelihood of DBM occurrence, the study delves deeper into the dynamics of DBM. Figure 1 shows the conceptual framework for the determinants of DBM among mother–child dyads.

This research study contributes to the understanding of the double burden of malnutrition (DBM) among mother–child dyads in India. While previous studies have often focused on either undernutrition or overnutrition, this study specifically aims to address the coexistence of both issues within the same households and explores the associated factors and inequalities. Wagstaff Decomposition analysis allows us to quantify the contribution of different socio-economic determinants to the observed income-related inequality in DBM. It helps understand which factors drive the inequality in DBM prevalence. Most nationally representative studies have focussed their attention on either undernutrition or over-nutrition. There has been little attempt to combine these two. It is crucial to understand the prevalence of wealth-based disparities in malnutrition in India to achieve equality, the central idea of the Sustainable Development Goals (SDGs). This is especially crucial in a country like India, where, progress made on health and development fronts has been highly inequitable and sporadically distributed. The primary research question of the paper is to understand the prevalence of the double burden of malnutrition among mother–child dyads in India and to assess the wealth-based inequalities in its prevalence.

## Data and methods

### Data

This study utilizes data from the fifth wave of the National Family and Health Survey (NFHS-5), a nationally representative survey conducted in the year 2019–21 under the stewardship of the Ministry of Health and Family Welfare (MoHFW), Government of India. International Institute for Population Sciences (IIPS), Mumbai, has been the nodal agency for surveying all the rounds of NFHS. This survey provides crucial information on reproductive and child health and women's autonomy, women and children's nutrition-related indicators, etc., aligned with various Sustainable Development Goals (SDGs) covering all 28 states and eight union territories. A two-stage, stratified cluster sample of 30,456 primary sampling units was constructed. The survey is an integrated multi-level survey of households, men and women in their reproductive ages, and biomarkers. A detailed description of sampling design and data collection methods are provided elsewhere (IIPS & ICF, 2021). Children under the age of 3 years and their mothers were considered eligible for this study. Out of the total eligible sample of 135,119 individuals, 8,781 pregnant women and those with missing information on Body Mass Index (BMI) (3416) were excluded from the study. Thus, the analytical sample for the study purpose was reduced to 122,922 dyads.

### Description of the variables

The double burden of malnutrition is employed as the dependent variable in this study. A household was classified to have the double burden of malnutrition if the mother was overweight/obese and the child was either stunted/wasted/underweight. Further, cases where “mother was not overweight/obese but child was stunted/wasted/underweight” or “mother was overweight/obese but the child was neither stunted/wasted/underweight” or “mother was  underweight/normal BMI and child were not malnourished” were considered as “without having the double burden of malnutrition”.

Anthropometric data on height and weight, and waist circumference collected in the survey were used to measure the nutritional status of young children and mothers. Mother's and children's (aged 24 months and above) weight was measured with an electronic SECA 874 flat scale, while their height was measured with a SECA 213 stadiometer. Children younger than age 24 months were measured lying down (recumbent length using a Seca 417 infantometer) while standing height was measured for the older children. Children whose height-for-age Z-score is below minus two standard deviations (− 2 SD) from the median of the reference population are considered short for their age (stunted). Children whose Z-score is below minus two standard deviations (− 2 SD) from the median of the reference population are considered thin (wasted). Children whose weight-for-age Z-score is below minus two standard deviations (− 2 SD) from the median of the reference population are classified as underweight. The variables (stunting, wasting, underweight) are categorized as binary: “1” for undernourished, indicating stunted/wasted/underweight and “0” for healthy, indicating not stunted /not wasted/not underweight children. BMI for mothers was calculated using the formula: weight in kilograms/height in meters squared. The BMI values for mothers were categorized as underweight if their BMI was below 18.5, normal or healthy if their BMI ranged between 18.5 and 24.9, overweight if their BMI ranged from 25.0 to 29.9, and obese if their BMI was 30.0 and above.

The study investigated whether the following socio-economic and demographic characteristics of mothers and children were associated with the risk of the double burden of malnutrition. The child characteristics included the sex of the child (male or female), age of the child (less than 18 months and 18 months and above), and birth order (1, 2–3, and 4 and above). The mother was asked if the child had fever, cough, and diarrhoea in the last two weeks and their responses were recorded as yes or no. The respondents were asked if the delivery was by caesarean section and their responses were categorized as yes and no. Further, the mothers were asked if they were currently breastfeeding, and their response was recorded as yes and no. the sex of the household head was categorized as male and female and the household size was grouped into 4 or less members, 5–6 members, and seven and above members. The ‘mother’s characteristics included ‘mother’s age (< 24 years, 25–29, 30–34 and 35–39, 40–44 and 45–49), educational attainment (No education, primary, secondary, and higher), place of residence (rural and urban), BMI (underweight, normal, overweight/obese). The other socio-economic variables included wealth index (categorized as poorest, poorer, middle, richer, richest), water source (improved, unimproved), religion (Hindu, Muslim, Others), caste group (SC, ST, OBC, and Others), and region (North, Central, East, North-East, West, and South). Abdominal obesity was characterized based on measured waist circumferences. Waist circumference is often used as a surrogate marker of abdominal fat mass^31^. Waist and hip circumferences were measured in centimetres using a Gulick tape according to standard protocols. Women were categorized as having abdominal obesity if they had a waist circumference ≥ 88 cm.

### Methods

Descriptive statistics were used to understand the sample distribution and to find the preliminary results. Bivariate analysis enabled to investigate the relationship between the double burden of malnutrition and several socio-demographic variables. Further, logistic regression was used to identify the factors affecting the double burden of malnutrition. Wagstaff decomposition analysis was applied to quantify the contribution of each inequality in the social determinants on the observed income-related inequality in the dual burden of malnutrition. The analysis is based on concentration curve (CC) and Concentration Index^32^ which have been used to determine the inequalities in the dual burden of malnutrition where CC denotes the cumulative share of malnutrition burden accounted for cumulative percentage of the individuals ranked by wealth index. The CI is computed as the twice the covariance of the dual burden of malnutrition and individuals wealth index, divided by the mean of the dual burden of malnutrition (Kakwani et al.^33^).

1 $$CI = \frac{2}{\mu } Cov \left( { Y_{i , } R_{i } } \right)$$

where ${\gamma }_{i }and {r}_{i}$ are the malnutritional status and fractional rank (in terms of the index of economic status) of the ith individual, respectively; μ is the mean of the malnutritional status.

The CI is decomposed into the contributions of k social determinants, where each contribution is calculated by averaging the health outcome variable’s sensitivity to each determinant and the level of wealth-related inequality in that component. Equation ^2^ demonstrates that the total wealth disparity in health preventive measures is composed of two parts: a ‘unexplained’ and deterministic (or ‘explained’) part.

$$CI = \mathop \sum \limits_{k} \left( { \frac{{\beta_{k} x_{k} }}{\mu }} \right)C_{k } + \frac{{GC_{e} }}{\mu }$$

The coefficient from regressing the health result on determinant k is ${\beta }_{k}$ in the first component. A category with a high (low) coefficient may have a relatively low (high) elasticity if the category has a low (high) frequency. The elasticity is calculated by weighing the coefficients by the frequency of the determinant using the mean of the determinant k (${x}_{k}$) and the mean of the outcome ($\mu$). When compared to the CI of the result, C_k_ is the concentration index for each of the k determinants. The elasticity describes the relationship between a change in the health dependent variable and a change in the explanatory k variable. The generalised CI (${GC}_{e}$) for the error term in the second component represents the amount of inequality that cannot be explained by the chosen components.

All statistical analyses were conducted using Stata version 16.

### Patient and public involvement

As the study utilized the nationally representative NFHS 2019-21 data, no patients or the public were involved in this research. The data is freely accessible through the web link: https://dhsprogram.com/data/dataset/India_Standard-DHS_2020.cfm?flag=1.

## Results

Table 1 shows the background characteristics of the study participants. Of the total eligible sample, around one-fifth of the mothers were underweight and a similar proportion was overweight/obese. Around one-third (32%) of the children were stunted, two-fifths (20%) were wasted and 30% were underweight. Around 48% of the child were either stunted/wasted/underweight and classified as undernourished. Around one-fifths of the birth occurred through C-section and most mothers were currently breastfeeding (80%). The majority of the households were headed by males (85%), had an improved source of drinking water (96%), seventy-four percent resided in rural areas, belonged to poorest wealth quintile (24%), and a majority had a secondary level of schooling (52%). Most of the respondents were in the age group 15–29 years, and the distribution of children by sex was almost same. Around 50% of the children were of second and third birth order. Around 14% of the children suffered from fever and cough in the last two weeks of the survey, and 9% had diarrhea recently.

**Table 1:** Background characteristics of the study population, NFHS-5.

Background characteristics	Unweighted frequency	Weighted percentage
Mother’s BMI		
Underweight	24,679	20.83
Normal	77,878	61.5
Overweight/Obese	20,365	17.66
Child stunted		
No	83,720	68.18
Yes	39,202	31.82
Child wasted		
No	99,524	80.49
Yes	23,398	19.51
Child underweight		
No	87,307	70.24
Yes	35,615	29.76
Child nutritional status		
Under-nourished	59,185	47.95
Healthy	63,737	52.05
Delivery by C-section		
No	96,937	76.4
Yes	25,985	23.6
Currently breastfeeding		
No	21,905	18.74
Yes	101,017	81.26
Region		
North	22,821	13.52
Central	31,188	27.16
East	23,909	26.44
North-east	18,378	3.79
West	10,945	12.34
South	15,681	16.75
Residence		
Urban	24,849	26.48
Rural	98,073	73.52
Wealth index		
Poorest	32,267	23.62
Poorer	28,474	21.57
Middle	24,163	19.79
Richer	21,108	18.86
Richest	16,910	16.16
Educational attainment		
No education	24,403	19.13
Primary	14,803	11.51
Secondary	64,985	51.93
Higher	18,731	17.42
Mother’s current age (in years)		
15–24	45,925	39.66
25–29	46,473	37.99
30–34	21,042	15.97
35–39	7538	5.2
40–44	1575	0.98
45–49	369	0.2
Child sex		
Male	64,130	52.29
Female	58,792	47.71
Age of the child		
18 months or less	66,041	53.3
More than 18 months	56,881	46.7
Birth order		
1	46,081	38.13
2–3	60,971	50.17
4 and above	15,870	11.71
Caste		
SC	25,034	23.27
ST	24,881	10.02
OBC	46,957	43.28
Others	26,050	23.43
Religion		
Hindu	90,594	79.57
Muslim	17,400	16.06
Others	14,928	4.38
Sex of household head		
Male	104,187	84.76
Female	18,734	15.24
Number of household's member		
1–4	30,263	24.09
5–6	46,460	37.54
7 and above	46,199	38.37
Sources of drinking water		
Unimproved	8015	4.17
Improved	108,131	95.83
Had fever in last 2 weeks		
No	106,047	85.32
Yes	16,800	14.68
Had cough in last 2 weeks		
No	105,725	85.33
Yes	17,067	14.67
Had diarrhea recently		
No	112,671	91.12
Yes	10,120	8.88
Abdominal obesity		
Abdominally non-obese	104,931	84.37
Abdominally obese	17,835	15.63

Table 2 presents the results from bivariate analysis and logistic regression estimates of having a dual burden of malnutrition in surveyed households. Chi-square test indicated a significant relationship between the double burden of malnutrition and background variables such as delivery by C-section, currently breastfeeding, region, residence, wealth, education, respondents’ age, child sex, age of the child, birth order, caste, religion, number of household members, water source and child's recent history of cough and diarrhea and maternal abdominal obesity. Overall, seven percent of the households had a dual burden of malnutrition i.e the mother was overweight/obese and the child was either stunted/wasted/underweight. The prevalence of double burden of malnutrition was higher among households where the child was born through C-section (12% vs. 6%), in southern states (12%), in richest wealth quintile households (12%), and mother had higher educational attainment (10%). Moreover, the dual burden of malnutrition was highest among households with mothers aged 35–39 years and children older than 18 months. Moreover, it was more profound among higher order births, the household headed by males, larger household sizes, and abdominally obese mothers (24% vs. 4%).

**Table 2:** Results of Bivariate and Logistic regression analysis: Prevalence and Risk factors of dual burden of malnutrition by socio-demographic characteristics, NFHS-5.

Background characteristics	Prevalence	Odds ratios (CI)
Delivery by C-section	Pr = 0.000	
No	5.71	®
Yes	11.65	1.31*** (1.24–1.39)
Currently breastfeeding	Pr = 0.000	
No	8.99	®
Yes	6.68	1.05* (0.99–1.12)
Region	Pr = 0.000	
North	6.44	®
Central	6.22	1.14*** (1.05–1.23)
East	4.83	0.99 (0.9–1.08)
North-east	4.35	1.06 (0.96–1.18)
West	8.68	1.48*** (1.34–1.62)
South	12.17	1.54*** (1.41–1.67)
Residence	Pr = 0.000	
Urban	10.73	®
Rural	5.81	1.09*** (1.03–1.16)
Wealth	Pr = 0.000	
Poorest	3.16	®
Poorer	5.5	1.39*** (1.27–1.52)
Middle	7.67	1.65*** (1.51–1.81)
Richer	9.45	1.7*** (1.54–1.88)
Richest	11.62	1.7*** (1.52–1.91)
Educational attainment	Pr = 0.000	
No education	4.87	®
Primary	5.78	1.06 (0.96–1.17)
Secondary	7.16	1.11** (1.02–1.2)
Higher	10.3	1 (0.9–1.1)
Mother’s current age (in years)	Pr = 0.000	
15–24	4.69	®
25–29	7.43	1.27***(1.19–1.36)
30–34	10.33	1.48*** (1.37–1.6)
35–39	12.21	1.59*** (1.44–1.77)
40–44	11.5	1.83*** (1.52–2.2)
45–49	15.61	1.55** (1.04–2.31)
Child sex	Pr = 0.000	
Male	7.43	®
Female	6.76	0.86*** (0.82–0.9)
Age of the child	Pr = 0.000	
18 months or less	6.43	®
More than 18 months	7.89	1.19*** (1.13–1.25)
Birth order	Pr = 0.000	
1	6.17	®
2–3	7.77	1.14*** (1.08–1.21)
4 and above	7.36	1.25*** (1.13–1.38)
Caste	Pr = 0.000	
SC	6.5	®
ST	3.56	1.02 (0.95–1.09)
OBC	7.45	0.91** (0.83–0.99)
Others	8.6	0.98 (0.91–1.04)
Religion	Pr = 0.000	
Hindu	6.5	®
Muslim	9.46	1.39*** (1.3–1.49)
Others	9.58	1.17*** (1.07–1.28)
Sex of household head	Pr = 0.189	
Male	7.16	®
Female	6.86	0.94* (0.88–1.01)
No of household members	Pr = 0.000	
4 or less	7.53	®
5–6	7.44	1.09*** (1.02–1.17)
7 and above	6.53	1.12*** (1.05–1.18)
Source of drinking water	Pr = 0.000	
Unimproved	4.55	®
Improved	7.23	1.08 (0.96–1.22)
Had fever in last week	Pr = 0.422	
No	7.19	®
Yes	6.59	1.09** (1.01–1.19)
Had cough in last 2 weeks	Pr = 0.001	
No	7.27	®
Yes	6.11	0.88*** (0.81–0.95)
Had diarrhea recently	Pr = 0.000	
No	7.25	®
Yes	5.62	0.96 (0.87–1.05)
Abdominal obesity	Pr = 0.000	
Abdominally non-obese	3.96	®
Abdominally obese	24.17	5.77*** (5.48–6.08)
	7.12	(cons_) 0.01 (0.01–0.02)

***Significant at 99% confidence level, ** significant at 95% confidence level, * significant at 90% confidence level.

® Reference Category.

Results from logistic regression analysis show that households, where births occurred by C-section were 1.31 times more likely to have a dual burden of malnutrition [OR: 1.31, C.I. 1.24–1.39]. Surprisingly, currently breastfeeding women were at higher risk of having a dual burden of malnutrition than women not currently breastfeeding [OR: 1.05, C.I. 0.99–1.12]. Moreover, it was found that households from the southern, western, and central parts of the country were at higher odds of the dual burden of malnutrition in comparison to the northern region [{OR: 1.54, C.I. 1.41–1.67}; {OR: 1.48, C.I. 1.34–1.62}; {OR: 1.14, C.I. 1.05–1.23}]. As we moved from the poorest to the richest wealth quintile households, the risk of having a dual burden of malnutrition increased significantly. We witnessed a higher risk of the dual burden of malnutrition among households where women had a secondary level of education; the child was aged more than 18 months, higher order births, increased women’s age, male-headed household, and increasing household members than their respective counterparts. To our surprise, we found that children who had a fever in the last 2 weeks were 1.09 times higher odds of having a dual burden of malnutrition than children who did not report having fever [OR :1.09, C.I. 1.01–1.19] and having a cough reduced the risk of having the dual burden of malnutrition [OR: 0.88, C.I. 0.81–0.95]. Abdominally obese women were 5.77 times more likely to have a dual burden of malnutrition than their counterparts [OR: 5.77, C.I. 5.48–6.08].

Table 3 shows the concentration index and the relative contributions of each determinant in the total wealth-based inequality in the double burden of malnutrition among mothers and children in India. The value of the contribution indicates the extent of inequality contributed by the explanatory variable. Additionally, the percentage contribution provides the relative contribution of each determinants to the observed inequality. A higher percentage contribution of the determinants means that the respondents with the characteristic in question were highly represented among the rich, and vice versa. The overall concentration index (0.24, *p* < *0.01*) is positive, suggesting that the inequality in DBM is pro-rich and that there is a higher concentration of DBM among the economically well-off sections of the population.

**Table 3:** Decomposition of the concentration index of the dual burden of malnutrition, NFHS-5.

	Elasticities		Concentration indices		Contribution	Percentage contribution
Delivery by C-section						
No						
Yes	0.005		0.278		0.005	8.571
Currently breastfeeding						
No						
Yes	0.006		− 0.044		− 0.001	− 1.681
Region						
Central						
North	− 0.002		0.300		− 0.002	− 3.866
East	− 0.003		− 0.314		0.004	6.387
North-east	− 0.001		− 0.319		0.001	1.513
West	0.002		0.221		0.002	3.025
South	0.005		0.281		0.005	8.571
Residence						
Urban						
Rural	− 0.010		− 0.158		0.006	10.420
Educational attainment						
No education						
Primary	0.001		− 0.259		− 0.001	− 1.176
Secondary	0.006		0.043		0.001	1.681
Higher	0.002		0.493		0.003	5.210
Mother’s current age (in years)						
15–24						
25–29	0.003		0.048		0.001	1.008
30–34	0.003		0.078		0.001	1.849
35–39	0.002		− 0.001		0.000	0.000
40–44	0.000		− 0.131		0.000	− 0.168
45–49	0.000		− 0.293		0.000	− 0.336
Child sex						
Male						
Female	− 0.004		− 0.003		0.000	0.000
Age of the child						
18 months or less						
More than 18 months	0.005		0.002		0.000	0.168
Birth order						
1						
2–3	0.006		− 0.002		0.000	0.000
4 and above	0.002		− 0.334		− 0.003	− 4.202
Caste						
OBC						
SC	0.000		− 0.115		0.000	0.000
ST	− 0.001		− 0.365		0.002	2.857
Others	0.000		0.172		0.000	0.000
Religion						
Hindu						
Muslim	0.004		0.008		0.000	0.168
Others	0.000		0.179		0.000	0.504
Sex of household head						
Male						
Female	− 0.001		− 0.057		0.000	0.168
No of household members						
7 and above						
4 or less	0.001		− 0.032		0.000	− 0.168
5–6	0.001		− 0.009		0.000	0.000
Source of drinking water						
Unimproved						
Improved	0.005		0.015		0.000	0.504
Had fever in last week						
No						
Yes	0.001		− 0.065		0.000	− 0.336
Had cough in last 2 weeks						
No						
Yes	− 0.001		− 0.052		0.000	0.504
Had diarrhea recently						
No						
Yes	− 0.001		− 0.095		0.000	0.336
Abdominal obesity						
Abdominally non-obese						
Abdominally obese	0.029		0.301		0.035	58.487

**Figure 2:**
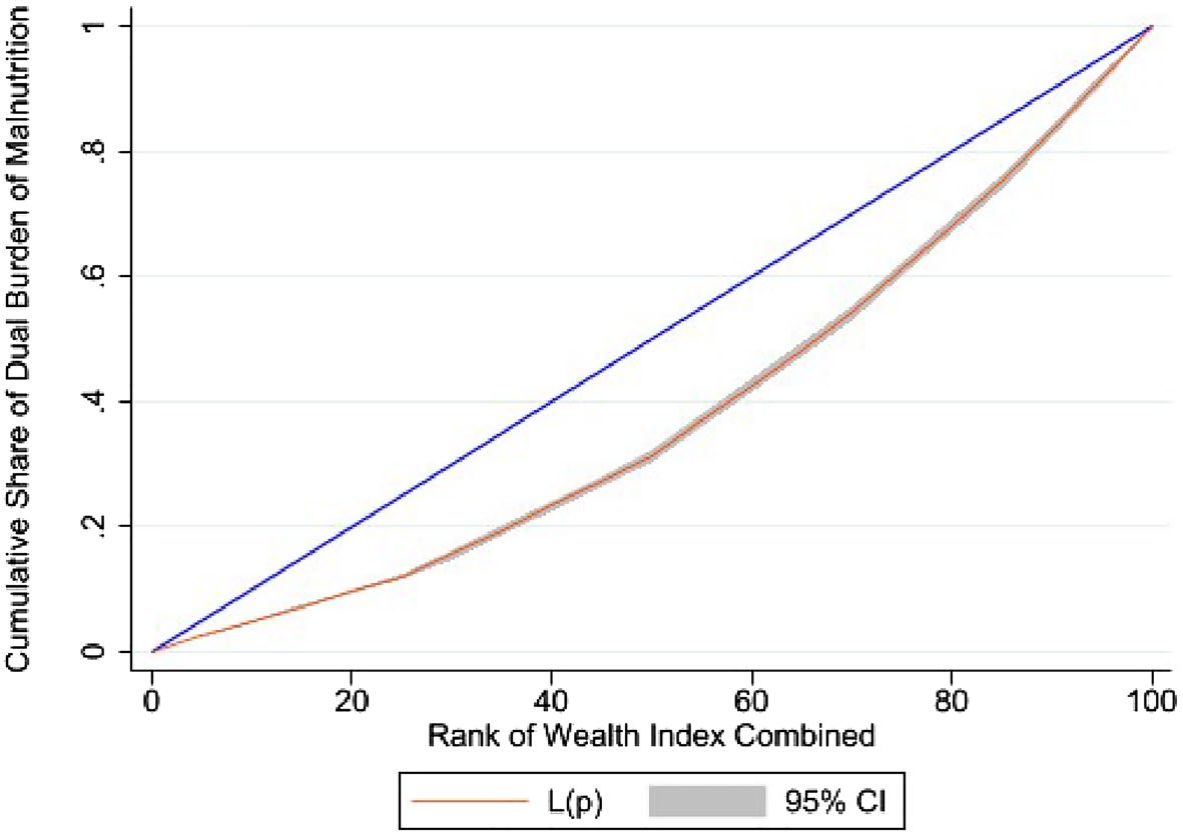
The concentration curve of income-related inequality in double burden of malnutrition, NFHS-5.

Most of the determinants exhibit a high level of inelasticity, meaning that their contribution patterns remain stable and unaffected by potential policy changes. However, abdominal obesity stands out as the determinant that shows a different pattern, suggesting that it could have a more responsive impact on changes in dual burden of malnutrition. Wagstaff decomposition analysis show that abdominal obesity is the most significant determinant of wealth-based inequality in DBM, contributing to nearly 3/5^th^ of the observed inequality with the burden falling on the rich. The next most important contributor to the inequality is geographical regions with its percentage contribution to the total inequality being nearly 16%, followed by the place of residence (10.4%). Other important contributors are births by caesarean section (8.6%), maternal education (5.7%), birth order (4.2%), and caste (2.9%). It is important to note that the percentage contributions of other crucial socio-demographic variables such as maternal age is pretty low (2.4%). The findings also indicated that there is an unexplained portion of differences in prevalence of dual burden of malnutrition, which cannot be accounted by the factors studied. Figure 2 presents the concentration curve of income-related inequality in double burden of malnutrition. It is evident that a large income-related gap persists in the distribution of DBM.

## Discussion

Child undernutrition under the age of five is a serious public health issue in India^34^. The Indian population's nutritional status varies greatly, with certain individuals experiencing both extraordinarily high rates of undernutrition in childhood (19% wasted, 36% stunted and 32% underweight) and overnutrition (12–46%). According to NFHS-5 data (2020–21), 24% of women (15–49 years old) are overweight or obese. Since, the childhood malnutrition is primarily influenced by a number of socioeconomic (religion, caste, education, wealth, family income), demographic (mother’s age at marriage), proximal (gestational age, birth interval, maternal BMI, birth interval), and environmental variables^35–37^, we first aimed to understand the distribution of double burden of malnutrition by various socio-demographic characteristics.

While India has made remarkable progress in curtailing undernutrition rates in the past decade, its prevalence is still alarmingly high. Additionally, owing to epidemiological transition, the rising prevalence of overweight and obesity is creating further strains on the health systems in India. The prevalence of double burden of malnutrition was found to be around seven percent among mother–child dyads. A study from India reports the prevalence of double burden of malnutrition among mother–child pairs to be 6 percent^38^, which is lower than the prevalence in Nepal^39^ and Bangladesh^40^.

Findings from regression analyses highlight a set of factors associated with an increased risk of DBM. The most important ones among them are: having a C-section birth, belonging to affluent households, currently breastfeeding, having high maternal age, and having a larger household size. These findings have been endorsed by previous studies as well. Similar to the present study, previous research also states that mothers with a C-section delivery are at a higher risk of having DBM than mothers with normal deliveries. The reason cited for the above is that women who deliver through C-section delay the initiation of breastfeeding and the cessation of breastfeeding also happens early^41,42^. Additionally, late breastfeeding initiation due to C-section hinders child growth and nutrition outcomes^42^.

In our study, belonging to wealthy households was a risk factor for DBM. This finding supports previous literature stating that mother–child pairs from richer households are more prone to having DBM^40^. The primary cause behind this is the dietary transition that happens with increased household wealth^43^. This is followed by an increase in the consumption of energy-dense foods that lack any nutrient value, and are obesogenic^44,45^. Previous studies have also stated that mothers aged 35 years or above have higher odds of having DBM^39,40^. These studies reveal that women in higher age groups were more prone to being overweight or obese than their younger counterparts.

Another exciting finding from the study is that maternal education exacerbates the risk of the double burden of malnutrition. This paradoxical finding could be attributed to the high prevalence of obesity among educated women^46^. Several previous studies have noted that the prevalence of obesity among educated women is higher than their non-educated counterparts^47^. One of the prime reasons behind this is that educated women are more likely to engage in non-manual occupations, thus, further reducing physical activity and contributing to a rise in overweight and obesity^6^. However, another set of studies states that the relationship between maternal education and overweight/obesity is complex and varies from country to country^48^. This could be explained by the fact that education alone may not lead to women adopting healthy behaviour for themselves and their children. Poor health and nutrition knowledge may cause women to be less mindful of making intelligent food choices regarding availability, accessibility, and cost^49^.

It is crucial to understand that while economic developments and improvements in socio-economic status play a role in reducing undernutrition rates, they also cause a concomitant increase in overweight/obesity^19,24^. Recent estimates suggest that improvements in socio-economic status contributed to a reduction of 29 percent in underweight and an increase of 46 percent in the prevalence of overweight/obesity between 2006 and 2016^24^. This is because, with increased income, dietary diversity increases, which is a positive change. On the other hand, consuming processed foods, saturated foods, take-home foods, and added sugars and salts increases^30^. Additionally, urbanization and economic growth are associated with more time spent at work and less time for leisure and physical activity^30^, further exacerbating the side effects of energy consumption on overweight/obesity and diet-related NCDs. Results indicated that households where women had abdominal obesity were at increased risk of having double burden of malnutrition. The predisposition of diabetes among women with abdominal obesity creates an intrauterine environment of insulin resistance and hyper-glycemia leading to fetal growth and childhood growth acceleration Maternal nutrition, intrauterine programming and consequential risks in the offspring^50^.

The issue of DBM among mother child dyads is not an isolated phenomenon; rather, it is influenced by the rapid increase in maternal Body Mass Index (BMI) and a slower rate of reduction in child malnutrition. These outcomes align with existing research conducted in Asia, where the coexistence of undernutrition and over-nutrition, referred to as double burden, has been observed^51^. It is widely acknowledged in the literature that the simultaneous occurrence of under- and over-nutrition is linked to the phenomenon of nutrition transition^8^. Recent studies underscore a swift change in the nutritional status of adults and evolving dietary preferences [34, 36], providing evidence of an ongoing nutrition transition in India. The shift towards a greater preference for energy-dense food items may lead to diminished nutrient intake for both adults and mothers during pregnancy, ultimately contributing to child undernourishment. This shift in dietary habits over the past decades could potentially account for the presence of DBM in India.

India has a sturdy policy framework to tackle malnutrition in all forms. Evidence-based nutrition interventions to address maternal and child malnutrition have existed for several decades. Programs such as the Integrated Child Development Scheme, Poshan Abhiyaan, National Health Mission, Mid Day Meal Scheme, and Targeted Public Distribution System have successfully adopted a holistic approach to tackling malnutrition in India by addressing several proximate and distal determinants of nutritional status in India. A flip side to the above is that most of these programs focus heavily on undernutrition, and other forms of malnutrition are somehow neglected. This is obvious, given historically high maternal and child undernutrition rates in India. The need of the hour is to shift this policy to focus on all forms of malnutrition, including undernutrition, over-nutrition, and micronutrient deficiencies.

The widespread inequalities should also be taken into account while framing policy interventions. Such programs commonly referred to as ‘double-duty’ ‘actions’, are potent enough for tackling multiple forms of malnutrition^51^. Such actions will also ensure that programs and policies targeted at reducing food insecurity and undernutrition are not unintentionally abetting the increase in the prevalence of overweight/obesity^51^. Additionally, incorporating holistic approach to reduce the risk of abdominal obesity that exacerbate the risk of DBM considering the social, economic and environmental factors might prove beneficial. Encouraging fortification of staple food with essential nutrients and educating individuals to make informed dietary choices might improve the nutritional status. Providing targeted nutrient supplementation to pregnant women, young children and Implementing labeling systems that provide clear and understandable nutritional information about food items will enable individuals and families to make healthier food choices.

Socioeconomic disparities, poverty, food insecurity, and unhealthy lifestyles, among other factors, collectively diminish the capacity to maintain metabolic balance within any given country. This, in turn, heightens the overall susceptibility to experiencing double burden of malnutrition (DBM) as well as other non-communicable diseases [6]. The phase encompassing a child’s initial 1000 days of life, often referred to as a critical window, necessitates focused attention, particularly in the context of mother–child pairs [4]. The transmission of malnutrition across generations, stemming from lower levels of maternal education, inadequate household infrastructure, insufficient breastfeeding practices, consumption of nutritionally deficient diets, and engagement in non-healthy lifestyle behaviors, provides avenues for potential interventions aimed at mitigating and addressing the DBM.

The present study has certain limitations that should be taken into cognizance. First, being based on cross-sectional data, the study could not establish a causal pathway between DBM and other explanatory factors. Second, the nutritional status of mothers and children was assessed using BMI only. BMI, as a measure of nutritional status, has several limitations. Despite being the most commonly used and inexpensive method, it is less accurate than other measures such as waist-hip ratio and skinfold thickness. Despite the limitations mentioned above, the study’s strength lies in the fact that it is based on a nationally representative large-scale dataset. This study provides evidence of the existence of DBM among mother–child pairs in India and the factors contributing to the same. It also provides insights into the presence of wealth-based inequality in the prevalence of DBM among mother–child pairs in India. This study, therefore, presents essential evidence for policy formulation and implementation pertaining to the health and nutrition of mothers and their children in India.

## Conclusion

It is crucial to understand that the malnutrition profile in India is evolving rapidly, with a rising prevalence of overweight/obesity along with staggering progress in the prevalence of undernutrition indicators. Additionally, this double burden of malnutrition is accompanied by widespread inequalities in its prevalence that have not been adequately addressed in India. The need of the hour is to develop double-duty actions and novel strategies to tackle the challenges posed by the double burden of malnutrition. Potential areas for intervention include promoting the production and consumption of various healthy food options and dissuading the marketing, production, and consumption of processed foods, snacks, and beverages. This can be done by strengthening the food systems to make them more affordable, diverse, and popular among the masses. Fortified food with essential nutrients and implementing labelling systems on food items providing nutritional information will enable making informed food choices. Further research should, therefore, focus on how double duty actions can be undertaken and promoted in the context of India's social, economic, and political backdrop.

## Data Availability

This study uses secondary data which is available on request ON DHS website https://dhsprogram.com/data/dataset/India_Standard-DHS_2020.cfm?flag=1.
